# Infection Strategies and Pathogenicity of Biotrophic Plant Fungal Pathogens

**DOI:** 10.3389/fmicb.2022.799396

**Published:** 2022-06-02

**Authors:** Johannes Mapuranga, Na Zhang, Lirong Zhang, Jiaying Chang, Wenxiang Yang

**Affiliations:** College of Plant Protection, Technological Innovation Center for Biological Control of Plant Diseases and Insect Pests of Hebei Province, Hebei Agricultural University, Baoding, China

**Keywords:** biotrophs, plant fungal pathogens, effectors, pathogen-host interaction, pathogenicity

## Abstract

Biotrophic plant pathogenic fungi are widely distributed and are among the most damaging pathogenic organisms of agriculturally important crops responsible for significant losses in quality and yield. However, the pathogenesis of obligate parasitic pathogenic microorganisms is still under investigation because they cannot reproduce and complete their life cycle on an artificial medium. The successful lifestyle of biotrophic fungal pathogens depends on their ability to secrete effector proteins to manipulate or evade plant defense response. By integrating genomics, transcriptomics, and effectoromics, insights into how the adaptation of biotrophic plant fungal pathogens adapt to their host populations can be gained. Efficient tools to decipher the precise molecular mechanisms of rust–plant interactions, and standardized routines in genomics and functional pipelines have been established and will pave the way for comparative studies. Deciphering fungal pathogenesis not only allows us to better understand how fungal pathogens infect host plants but also provides valuable information for plant diseases control, including new strategies to prevent, delay, or inhibit fungal development. Our review provides a comprehensive overview of the efforts that have been made to decipher the effector proteins of biotrophic fungal pathogens and demonstrates how rapidly research in the field of obligate biotrophy has progressed.

## Introduction

Plants are threatened by a variety of microorganisms during their growth, including biotrophs, necrotrophs, or hemi-biotrophs (Glazebrook, 2005). Different microorganisms use different strategies to infect and damage plants. Gratifying progress has been made so far in the pathogenesis of necrotrophic or hemi-biotrophic microbes, and the pre-infection pathways and pathogenesis of necrotrophic microbes have been identified. However, the pathogenesis of obligate parasitic pathogenic microorganisms is difficult because they cannot reproduce and complete their life cycle on artificial media. Biotrophic plant fungal pathogens are among the ten pathogens considered most important internationally to plant pathology (Dean et al., 2012). Obligate parasitic plant fungi are a large group among which rust and powdery mildew are the two largest groups. Rust fungi represent one of the most important fungal orders, consisting of more than 8,000 known species (Aime et al., 2014; Lorrain et al., 2019). These fascinating parasites attack a wide range of plants and cause severe damage (Lorrain et al., 2019). It is typical of many powdery mildews where the fungi form a powdery coating of white spores on the leaf surface and some common examples include powdery mildew of cereals and grasses (*Erysiphe graminis*) and gooseberry (*Sphaerotheca mors-uvae*) (Moore, 2020). The disease causes high yield losses of up to 30% (Hückelhoven, 2005).

All pathogenic fungi, despite their different infection and nutrient acquisition mechanisms, can be recognized by the defense system of the plant resulting in activation of the host defense system. A strong host response to fungal infection is facilitated by the activation of local and systemic responses due to innate immunity, over a prolonged period of time (Schwessinger and Ronald, 2012). The ability of plants to effectively respond to pathogen infection preliminarily depends on pre-formed mechanisms of defense, which include pre-formed barriers such as cuticle and phytoanticipins. Cuticle is an important barrier to pathogen penetration in which cell wall and cuticle thickness influence resistance against pathogens. In some plants, adult plant resistance is linked to the decreased ability of fungal pathogens to penetrate through the thicker and tougher cell walls (Guest and Brown, 1997). Cuticular wax is composed of cutin and wax which is deposited on the leaf surface hence water cannot be retained on it and this will complicate the germination of spores in the absence of water. Phytoanticipins act as pre-formed constitutive chemical barriers against microbial attack. Some of the phytoanticipins like glucosinolates and cyanogenic glycosides exist as inactive precursor stores in healthy tissues and are only activated as a result of tissue damage (Tiku, 2020). Pattern-recognition receptors (PRRs) localized on the host membrane recognize PAMPs (pathogen-associated molecular patterns) within the host apoplast during pathogen infection, which in turn activates PAMP-triggered immunity (PTI). Biotrophic plant fungal pathogens suppress PTI components by secreting virulence factors known as effectors into the host cells thereby causing diseases (Martel et al., 2021). In response, host plants evolved another layer of immunity in which the plant intracellular nucleotide-binding/leucine rich-repeat (NB-NLR) receptors and resistance (R) proteins specifically detect cognate avirulence factors and trigger a robust defense response called effector-triggered immunity (ETI). Direct or indirect effector recognition activates NRLs and this results in an array of induced mechanisms including reactive oxygen species (ROS) production, hypersensitive response, generation of phytoalexins, and accumulation of pathogenesis-related proteins (Amil-Ruiz et al., 2011). An integrated signaling network mediates the two-tier defense system and this system largely shares the downstream signaling machinery (Tsuda and Katagiri, 2010). How pathogens overcome the host’s plant two-tiered defense system is a hot topic in plant pathology (Harris et al., 2020). How the biotrophic plant pathogenic fungi can successfully infect the host and establish a parasitic relationship in which they can manipulate the host physiology and defense system is still the main area of work of many researchers worldwide.

The inability to culture or genetically modify obligate biotrophic fungal pathogens *in vitro* complicates the study of the molecular bases of rust fungal pathogenicity. With technological advances and the use of new experimental methods, transcriptome sequencing and metabolomics are increasing our understanding of infection strategies, the functions of biotrophic fungal effectors, and the mechanisms underlying pathogen-host interactions. Researchers have endeavored to discover the pathogenic mechanisms and in particular to study the functional characteristics of effectors secreted by biotrophic fungal pathogens through gene silencing, RNAi and overexpression, either in their host or in a heterologous system (Jaswal et al., 2020; Prasad et al., 2020). In this review, we describe the definitions, examples, and characteristic features of biotrophic fungal pathogens, and infection strategies of biotrophic plant fungi, the effectors of biotrophic fungal pathogens, and how they manipulate the plant immune system, and summarize the major research advances on biotrophic fungal pathogens.

## What Are Biotrophic Plant Pathogenic Fungi?

Apart from the use of the term biotroph in several fields of research, there is still no clear definition of this terminology. A biotroph is characterized by an exceptional lifestyle that supports nutrient acquisition from living host cells and is completely dependent on the host for successful completion of the life cycle (Stotz et al., 2014; Gebrie, 2016). Biotrophs are pathogenic organisms that are completely dependent on the host cells for their nutrient acquisition and can secrete effectors to suppress or regulate plant basal defense. They form some sophisticated structures such as appressoria, infection peg, and haustoria to facilitate infection of epidermal cells and hyphae for nutrient uptake without damaging the host cells and with different forms in different Eumycota (Mendgen and Hahn, 2002; Delaye et al., 2013). Their lifestyle has complicated the detailed analysis of the molecular mechanisms underlying their pathogenicity, i.e., host evasion or suppression of plant immunity (Duplessis et al., 2011). Biotrophs generally include fungus rusts (Basidiomycetes), powdery mildew pathogens (Ascomycetes), and Oomycetes (downy mildew and white rusts) as obligate biotrophs. The biotrophic plant fungal pathogens consist of the two major groups of rust and powdery mildew. Rust fungi are among the most serious pathogens of major crops worldwide such as wheat, barley, soybean, maize, millet, flax, as well as coffee, etc. (Fisher et al., 2012). These include *Puccinia striiformis* f. sp. *tritici* (*Pst*), *Puccinia graminis* f. sp. *tritici* (*Pgt*), and *Puccini triticina* (*Pt*) which cause stripe rust, stem rust and leaf rust on wheat respectively, *Melamspora lini* (*M. lini*) (which cause flax rust), *Phakospora pachyrhizi* (which cause Asian soybean rust), *Hemileia vastatrix* (which cause coffee leaf rust), and *Melampsora larici-populina*, which cause defoliating poplar leaf rust disease. All these pathogens cause high yield losses of more than 15–∼80% if not controlled (Lawrence et al., 2007; Kelly et al., 2015). Powdery mildew fungi are widespread and vital fungal pathogens that infect over 10,000 plant species worldwide and cause yield losses of up to 40% in various frugal crops such as wheat and barley, as well as vegetable crops and fruit trees (Takamatsu, 2014).

**FIGURE 1:**
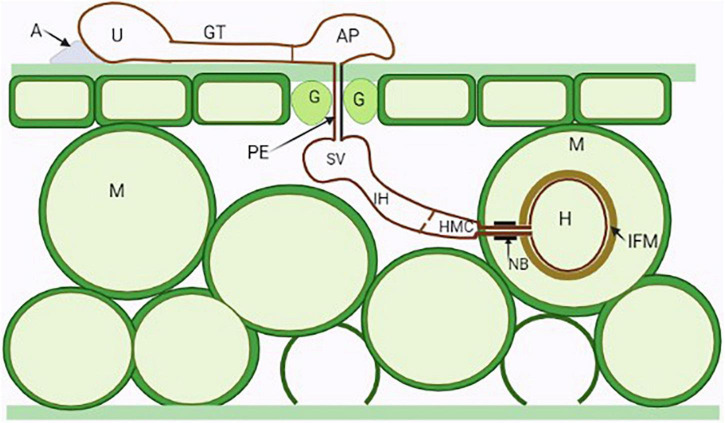
Illustration of the general infection process of a dikaryotic stage rust fungus. A urediospore (U) attached to an adhesion pad (A) germinate forming germ tubes (GT) which sense the topography of host cuticle and develop appressoria (AP) above stomata (Guard cells—G). Penetration occurs through stomata by the penetration hyphae (PE) into the substomatal spaces where the fungus differentiates into substomatal vesicles (SV) and elongates into an intercellular hypha (IH) which comes into contact with the host mesophyll cells (M) and develops haustorial mother cells (HMC). Following this, haustorial formation is initiated, neckbands (NB) are formed around the site of penetration of the mesophyll cell and an interfacial matrix (IFM) develop between the haustoria cell wall and the cell plasma membrane.

One of the key features of obligate biotrophic fungal plant pathogens is their full dependence on the living host plant tissues, that is, they can only feed, grow, and reproduce on their living host (Latijnhouwers et al., 2003; Voegele and Mendgen, 2011; Kemen et al., 2015; Dracatos et al., 2018; Tang et al., 2018). They are highly and irreversibly adapted to their host plant. This implies that, generally, obligate biotrophic fungal pathogens has been defined by their inability to grow on artificial media. They have narrow, specialized host ranges and they enter their host through natural openings or by direct penetration (Moore, 2020). Biotrophic fungal pathogens are characterized by specialized structures called the haustoria that serve for nutrient absorption, and secretion of effector proteins to the host cytoplasm *via* the extrahaustorial matrix (Figure 1). The extrahaustorial matrix, a gel-matrix enriched in proteins and carbohydrates from both the pathogen and the host, is essential in maintaining the biotrophic lifestyle including plant pathogen recognition evasion (Perfect and Green, 2001; Green et al., 2002; Caillaud et al., 2014; Oliveira-Garcia and Valent, 2015). Although hemi-biotrophic fungal pathogens form haustoria during their biotrophic phase, the haustoria of the biotrophic fungal pathogens have a neck ring that seals the interface between the host and pathogen plasm membrane, disconnecting the extrahaustorial matrix from the plant and fungal apoplast and establishing a biotroph-specific compartment (Kemen et al., 2015; Figure 1). It is a characteristic of biotrophic plant pathogens that they are able to alter and reprogram the host metabolism (Kleemann et al., 2012). The ability of these pathogens to secrete effector proteins *in planta* is crucial in this context. They possess interfacial layers comprised of carbohydrates and proteins that separate fungal and plasma membranes. The characterization of the biotrophic plant fungal pathogens is based the genetic analysis of disease resistance with plants, how defense against fungal pathogens is regulated, and is also based on genome derived analysis of carbohydrate-active enzyme gene content. They secrete a limited amount of lytic enzymes to promote successful penetration of the host cell without any toxin production (Kemen et al., 2015). Host viability is essential for biotrophic plant fungal pathogens. Although biotrophic plant fungal pathogens are ubiquitous and have different lifestyles, they share some important common characteristics (Table 1).

**TABLE 1:** Characteristics of the obligate biotrophic plant pathogenic fungi.

Feature	Host-pathogen interaction	References
Obligate, full dependency on the host and these pathogens cannot be cultured on artificial media	Only grow and reproduce in nature in association with living host plants and cannot be axenically cultured, for example rusts and powdery mildews	Scott, 1972; Kemen et al., 2015; Ni et al., 2016; Dracatos et al., 2018; Tang et al., 2018
Have a narrow host range with long-term suppression of host defense	Narrow, specialized host ranges for examples rusts, powdery mildews	Ni et al., 2016; Glazebrook, 2005; Lorrain et al., 2019
Secrete limited amounts of lytic enzymes	Secreted enzymes like cutinases, cellulases, and pectinases are key during germination and penetration to breach the plant surface	Mendgen and Hahn, 2002; Kemen et al., 2015
Possess highly sophisticated infection structures, such as appressoria, haustoria, carbohydrate-rich, and protein-containing interfacial layers that separate fungal and plasma membranes	Both rusts and powdery mildews develop sophisticated infection structures to facilitate penetration with minimal host plant damage	Mendgen and Hahn, 2002; Oliveira-Garcia and Valent, 2015; Pawlowski and Hartman, 2016
Specialized entry, e.g., direct (mechanical) entry or through natural openings	Direct penetration occurs in powdery mildews caused by *Blumeria* species and penetration through stomatal openings occurs in the rust fungi, like the *Uromyces fabae*	Hahn and Mendgen, 1997; Moore, 2020
Regulated by specific resistance genes which induce hypersensitive cell death in incompatible interactions and also trigger the SA-dependent defense pathways	Biotrophic fungal pathogen infection triggers *R* gene-mediated resistance, *SA* and *HR* signaling in response to infection confers resistance	Mendgen and Hahn, 2002; Hammond-Kosack and Parker, 2003; Belkhadir et al., 2004; Marone et al., 2013
Frequently survive on the host as dormant propagules and growth on the surface epidermic cells or intercellular of the host plant	Essential for attachment, adhesion and pathogenicity for fungal growth and development	Moore, 2020

## Biotrophic Plant Fungal Pathogens Infection Strategies

Biotrophic fungi employ various strategies to avoid triggering host defense responses. These include forming specialized infection structures to avoid host recognition, facilitating attachment, penetration, and propagation to successfully establish pathogenesis (Oliveira-Garcia and Valent, 2015).

### Formation of the Advanced Infection Structures and Limited Secreted Amounts of Lytic Enzymes

Organisms that cause rusts and powdery mildews employ either direct penetration or penetration through the stomatal openings from the appressorium. In plant-pathogen interactions, the ability of the fungal spore to adhere to the host plant is considered a fundamental requirement for spore germination, germ tube elongation, and appressorium formation (Tucker and Talbot, 2001). Most powdery mildew fungal species use their infection structures called appressoria to penetrate directly into cuticle and cell wall of the host plant to colonize exclusively the epidermal cells of the plant, whereas most rust fungi form their appressorium over the stomata to penetrate the intercellular spaces of the mesophyll, potentially bypassing epidermal defense responses. Chemical or physical signals from the host plant including ethylene signals (Sharma and Gautam, 2019), topographic signals (Jones et al., 2001), cutin monomers (Sharma and Gautam, 2019), and substratum hydrophobicity (Kamakura et al., 2002) trigger the formation of appressoria.

In wheat rust, for example, the dikaryotic urediospores germinate within a short time once they land on the wheat epidermis when the humidity meets the germination requirements and the germ tube extends perpendicular to the vein and is directed toward stomatal cells of the leaf surface (Figure 1). An appressorium then forms above the stomata, followed by the development of a penetration peg to invade the host (Voegele et al., 2009). The formation of the penetration peg is initiated by haustorial mother cells, from which some lytic enzymes are secreted to promote successful penetration of the host cell wall, followed by the differentiation of the haustorium in the host cells (Kim, 2015). The continued growth of the peg within the substomatal chamber penetrates the host tissues (Chethana et al., 2021), and the cavity forms a vesicle (substomatal vesicle), which in turn forms one or more infection hyphae. *Uromyces fabae* secretes cell wall-degrading enzymes (pectate lyases, pectin esterases, and cellulases) under strict progressive regulation, only after the formation of the haustorial mother cells or infection hyphae and appressoria formation (Deising et al., 1995). The sequential production of these enzymes is directly related to the extreme local requirements for host cell entry during haustorium formation inside the leaf (Mendgen and Hahn, 2002).

Rust fungi possess monokaryotic and dikaryotic forms, all of which produce haustoria during their infection process. Dikaryotic haustoria arise from haustorial mother cells and have a haustorial neck with a haustorial body associated with the intercellular hyphae (Harder and Chong, 1984). The dikaryotic nature of fungal rust implies that they harbor significant genetic variation that is shared between the two haplotypes (Figueroa et al., 2020). Monokaryotic haustoria are terminal intercellular hyphae that have a septum near the penetration site (Gold and Mendgen, 1984; Harder and Chong, 1984; Heath, 1995). A distinct dense-neckband separates the extrahaustorial matrix of the rust fungus from the apoplasm (Figure 1). The formation of haustoria induces significant re-organization of host cytoskeleton, nuclear DNA and endomembrane system (Kobayashi et al., 1994; Heath, 1997; Voegele and Mendgen, 2003). The haustoria are not only a sophisticated structure for nutrient acquisition, but also an intense site where all secreted proteins, including effectors, are expressed (Garnica et al., 2014), and it has also been speculated that they are essential for signal transduction, communication, and evasion of host recognition (Hahn and Mendgen, 2001; Voegele and Mendgen, 2003, 2011). This implies that any interaction in obligate biotrophs involves exclusive and specific differentiation of infection structures, and that the exact specialization that occurs is highly dependent on the host and pathogen in question (Van Sumere et al., 1957). The urediospores of the soybean rust fungus *Phakopsora pachyrhizi* unwaveringly penetrate the host epidermis and form a highly turgid appressorium (Goellner et al., 2010; Loehrer et al., 2014). The physical force exerted by *P. pachyrhizi* appressoria based on turgor pressure is 3.9 or 5.5 μN, the osmotic potential is 5.13 MPa, and sugar alcohols are the predominant osmolytes within these cells (Loehrer et al., 2014). The turgor pressure of glycerol creates the mechanical strength of the appressorium through the breakdown of lipid and glycogen stores within as well as melanin in the cell walls of the appressoria (Latijnhouwers et al., 2003; Loehrer et al., 2014).

Powdery mildew fungal pathogens infect the aerial parts of higher plants and rarely kill their hosts, but their pathogenesis causes major yield losses mainly due to withdrawal of nutrients from the host plant, reduction of photosynthesis, stunted growth, and increased respiration and transpiration (Wu et al., 2021). Landing of conidial spores on the host surface is followed by attachment and penetration into the host cuticle and cell wall. They also form some sophisticated structures such as the appressorium, which is a key structure for invasion (Hückelhoven, 2005), and the hook-shaped appressorium grows over the cell surface of the epidermis. Meanwhile, powdery mildew germ tubes penetrate directly the host plant cell wall using both enzymatic and mechanical power forces (appressoria) (Green et al., 2002). Cell wall-degrading enzymes of plant pathogenic fungi play a fundamental role during infection by disrupting the host cell defense system and thereby acting as virulence or pathogenicity factors in some plant-pathogen interactions (Wood, 1960; Köller et al., 1982). During *Bgh*-barely interactions, *Bgh* accumulates 3-hydroxykynurenine, a redox-active substance that facilitates cross-linking of the pathogen to its host surface (Wilson et al., 2003). *Bgh* also expresses a secreted catalase that is essential in the removal of hydrogen peroxide produced by the host to cross-link its cell wall for penetration resistance, promoting invasive growth and spread during host infection (Zhang et al., 2004). Successful penetration results in the development of a penetration peg that penetrates directly into the epidermal cuticle of the host plant leading to the formation of haustoria which invaginates the host plasma membrane and are important for nutrient acquisition without damaging the host. The haustoria of powdery mildew formed in the epidermal cells of the host are ultimate unicellular structures consisting of a globular central body with projecting filamentous lobes (Gil and Gay, 1977).

Biotrophic plant pathogenic fungi can also attack their host by modulating pH which creates a favorable environment for infection, thus promoting pathogenicity (Strange, 2007). In addition, biotrophic pathogenic fungi produce some secondary metabolites such as siderophores, and pigments that promote their virulence and can also produce or modify hormones of the host plant such as; auxins (IAA), gibberellic acids (GA), jasmonic acid (JA), cytokinins, and abscisic acid (ABA), thus disrupting the corresponding plant hormone signaling pathways (Patkar et al., 2015; Shen et al., 2018). Wheat stem rust fungus specifically produces tryptophan 2-monooxygenase in the sucker, which causes the wheat cells to produce a large amount of indole acetic acid, which in turn regulates host disease resistance and promotes pathogen infection (Yin et al., 2014; Prasad et al., 2019). In research on the pathogenicity of *Pt*, adenylate cyclase (AC), a signaling molecule, was found to influence the pathogenicity of the leaf rust pathogen (Panwar et al., 2013).

### Secreted Effector Proteins

Invading pathogens also require some sophisticated strategies to overcome the host defense system. For successful infection and establishment of compatible interactions leading to proliferation, biotrophic fungi must counteract PTI (Dangl and Jones, 2001) to evade the host defense system, and to facilitate this, the pathogen secretes several effector proteins. The pathogen’s effector proteins are distinctive recognition targets for the host defense system, whereas components of the host defense signals or their receptors are the main target of effectors during infection. Currently, effectors are considered as a group of proteins without mutual conservation, that are uniquely responsible for some crucial functions such as tethering the host defense machinery by producing cytotoxicity to facilitate pathogen progression (Jaswal et al., 2020). After invasion of the host, effectors suppress the host’s basal defense and manipulate its physiology to promote their invasive growth and multiplication. Several biotrophic fungal effectors exploit this mode of action by sequestrating chitin and also make use of other various mechanisms to subvert PTI (Table 2). Effector genes are subject to high selection pressures, and therefore evolve rapidly, giving them a particular mode of action that allows them to manipulate the host’s defense system and physiology. For a strong defense system against invading pathogens, the host harbors *R*-genes that recognize pathogen effectors and transforms a virulent pathogen into an avirulent one (Jones and Dangl, 2006). Avirulence (*Avr*) genes encoded effector proteins are recognized by host *R* genes (Flor, 1971). However, in various pathosystems there is an indirect interaction between *R* and *Avr* gene products and this follows a guard or decoy model (Jones and Dangl, 2006).

**TABLE 2:** Biotrophic plant fungal pathogens effectors involved in the manipulation of host reaction.

Pathogen	Effector protein	Host	Localization	Function in virulence	References
*Pgt*	PgtSR1	Wheat	Unknown	Suppresses RNA silencing in plants and impedes basal plant defense by altering the abundance of small RNAs that serve as defense regulators	Yin et al., 2019
	AvrSr35	Wheat	Colocalize in the ER	Suppresses cell death signaling activities	Salcedo et al., 2017
	AvrSr50	Wheat	Cytosol and nucleus	Suppresses cell death signaling activities	Chen et al., 2017
	AvrSr27	Wheat	Unknown	Suppresses cell death signaling activities	Upadhyaya et al., 2021
*Pst*	Pst_4	Wheat	Cytoplasm	Disrupts sorting of chloroplast protein TaISP thereby suppressing host ROS accumulation	Wang et al., 2021
	Pst_5	Wheat	Cytoplasm	Disrupts sorting of chloroplast protein TaISP, thereby suppressing host ROS accumulation	Wang et al., 2021
	PSEC2	Wheat	Cytoplasm and nucleus	Suppresses PTI-related callose deposition	Su et al., 2021
	PSEC17	Wheat	Cytoplasm and nucleus	Suppresses PTI-related callose deposition	Su et al., 2021
	PSEC45	Wheat	Cytoplasm and nucleus	Suppresses PTI-related callose deposition	Su et al., 2021
	PSTG_10917	Wheat	Chloroplast	Capable of halting programmed cell death	Ozketen et al., 2020
	PNPi	Wheat	Apoplast	Interaction with host TaPR1a and reduces host defense responses	Bi et al., 2020
	PstCEP1	Wheat	Cytoplasm	Suppresses programmed cell death	Tao et al., 2020
	Pst18363	Wheat	Cytoplasm	Suppresses ROS accumulation by interacting with wheat Nudix hydrolase 23 TaNUDX23	Yang et al., 2020
	PstGSRE1	Wheat	Cytoplasm	Suppresses host PTI-associated callose deposition and hydrogen peroxide accumulation	Qi et al., 2019
	PST_12806	Wheat	Chloroplast	Essential for full virulence. Interacts with Rieske domain in the C-terminal of host TaISP protein	Xu et al., 2019
	PST_8713	Wheat	Cytoplasm and nucleus	Suppresses host PTI-associated PCD and callose deposition	Zhao et al., 2018
	PSTha5a23	Wheat	Cytoplasm	Suppresses host PTI-associated PCD and callose deposition	Cheng et al., 2017
	PEC6	Wheat Barley	Cytoplasm and nucleus	Interacts with adenosine kinases (ADKs) with generic functions to suppress PTI	Liu et al., 2016
	PST02549	Wheat	Processing bodies	Participates in the uncapping, degradation, and storage of messenger RNA by forming protein complexes	Petre et al., 2016
*Pt*	Pt3 and Pt27	Wheat	Unknown	Function in avirulence against wheat leaf rust in resistant genotypes	Segovia et al., 2016
	Pt18906	Wheat	Nucleus and cytoplasm	Acts in the cytoplasm and may cause accumulation of reactive oxygen species and callose in TcLr10 + 27 + 31	Qi et al., 2020
*M. larici-populina*	124202	Poplar	Endomembrane	Participates in vesicle-mediated trafficking but is less likely to significantly suppress the plant defense system	Gaouar et al., 2016; De Guillen et al., 2019
	MLP37347	Poplar	Plasmodesmata	Interacts with glutamate decarboxylase 1 (GAD1). Promotes enhanced plasmodesmatal flux and reduces callose deposition	Rahman et al., 2021
*P. pachyrhizi*	PpEC23	Asian Soybean	Nucleus and Cytosol	Suppresses host immune responses by physically interacting with soybean transcription factor GmSPL121	Qi et al., 2016
*Bgh*	CSEP0027	Barely	Cytosol and nucleus	Interacts with *Hv*CAT1 to regulate host immunity regulation and probably ROS homeostasis to promote virulence during infection	Yuan et al., 2021
	CSEP0139	Barley	Cytosol and nucleus	Suppresses cell death and promotes virulence	Li et al., 2021
	CSEP0182	Barley	Cytosol and nucleus	Suppresses cell death and promotes virulence	Li et al., 2021
	BEC1019	Barley and wheat	Cytosol and nucleus	Essential for virulence Essential for haustorial formation	Zhang et al., 2019
	CSEP0081	Barley	Cytoplasm and nucleus	Essential for penetration and formation of haustoria	Ahmed et al., 2016
	CSEP0254	Barley	Cytoplasm and nucleus	Essential for penetration and formation of haustoria	Ahmed et al., 2016
	BEC1054	Barley	Cytoplasm	Interaction with barley PR5, eEFG1G, MDH, and zGST to promote *Bgh* virulence at different levels.	Pennington et al., 2016
	CSEP0105	Barley	Cytosol and nucleus	Essential for virulence Interferes with Hsp16.9 and Hsp17.5 chaperon activity	Ahmed et al., 2015
	CSEP0162	Barley	Cytosol and nucleus	Promotes virulence Interferes with Hsp16.9 and Hsp17.5 chaperones activity.	Ahmed et al., 2015
	BEC3	Barley	Cytosol and nucleus	Interferes with defense-associated host vesicle trafficking	Schmidt et al., 2014
	BEC4	Barley	Cytosol and nucleus	Interferes with defense-associated host vesicle trafficking	Schmidt et al., 2014
	BEC1011	Barley	Cytoplasm	Interferes with pathogen-induced host cell death	Pliego et al., 2013
	CSEP0055	Barley	Apoplast	Essential for virulence Interaction with PR17 and PR1 proteins	Zhang et al., 2012
*Bgt*	AvrPm2	Wheat	Cytoplasm and nucleus	Suppresses the recognition of *Avr*	Bourras et al., 2016
	SvrPm3^*a*1/*f*1^	Wheat	Cytoplasm and nucleus	Facilitates evasion of pathogen recognition by *R* genes	Bourras et al., 2016

Effectors are mostly expressed in the haustoria and some expressed in infection hyphae of obligate biotrophs and can be categorized according to their specific site of action in the host, i.e., apoplastic and cytoplasmic effectors (Kamoun, 2006; Tanaka et al., 2020). Effectors of biotrophic fungal pathogens are either delivered in the cytoplasm of the host plant where they unswervingly manipulate processes in cells of the host (cytoplasmic effectors), or they may remain in the apoplast space to protect fungal cells from host plant defense components (apoplastic effectors) (Tanaka et al., 2020). Apoplastic effectors, which are important for full virulence, can inhibit host plant enzymes such as the peroxidases, chitinases, and proteases that can be harmful to the pathogen. Cytoplasmic effectors are delivered directly into the host plant cytoplasm, either by infection vesicles or haustoria, which are specialized structures (Kamoun, 2006), and accumulate in a biotrophic interfacial complex, some of them subsequently targeting specific compartments of the plant cell. They are recognized by intracellular R proteins of the cytoplasmic nucleotide-binding site leucine-rich repeat (NBS-LRR) and activate HR, which curbs invasive growth of the pathogen (Jones and Dangl, 2006; Dodds and Rathjen, 2010). Biotrophic plant fungal pathogens have also evolved some sophisticated mechanisms to direct their effectors to the host chloroplast by mimicking host sorting signals (Kretschmer et al., 2019). Effectors target the chloroplast where they suppress host plant basal immunity by suppressing cell death, decreasing the expression of defense-related genes and callose deposition, and causing accumulation of ROS (Petre et al., 2015; Xu et al., 2019).

Some biotrophic fungal effector proteins function as RNAi suppressors and it has been speculated that conserved proteins are the main target of these suppressors so that they can deactivate the host RNAi machinery. For example, the *Pgt* effector PgtSR1 suppresses host RNA silencing and impedes basal plant defense by swaying the spread of small RNAs that function as regulators of host defense (Yin et al., 2019). Host RNAi suppression machinery leads to inadequate regulation of various host defense pathways facilitating successful infection. Degradation of host RNA by biotrophic fungal effectors also helps in manipulating the host defense machinery (Jaswal et al., 2020). Pst02549 accumulates in processing bodies (P-bodies) and is involved in the uncapping, degradation, and storage of messenger RNA through the formation of protein complexes, thus facilitating infection (Petre et al., 2016). Effectors of biotrophic fungi also bind directly to the host DNA promoter, thereby regulating or altering its transcriptional processes, resulting in defective defense genes (Ahmed et al., 2018). Moreover, the effectors of biotrophic fungi camouflage the host as modulators, leading to the diversion of the metabolic flux of many compounds, resulting in a deficiency of precursors or compounds responsible for host defense (Tanaka et al., 2014; van der Linde and Göhre, 2021). Some well-known other examples are: CSEP0055 (Zhang et al., 2012), BEC3, BEC4 (Schmidt et al., 2014), CSEP0105, CSEP0162 (Ahmed et al., 2015), BEC1054 (Pennington et al., 2016), BEC1019 (Zhang et al., 2019), CSEP0027 (Yuan et al., 2021), CSEP0139 and CSEP0182 (Li et al., 2021) from *Bgh* and AvrPm3^*a*1/*f*1^ from *Bgt* (Bourras et al., 2015; Parlange et al., 2015). The effector protein PNPi of *Pt* acts directly on wheat TaNPR1 to inhibit resistance of wheat to leaf rust (Bi et al., 2020). *AvrPm3*^*a*2/*f*2^ and *AvrPm2* of *Bgt* (Bourras et al., 2015, 2018), *Avr*_*a*1_, *Avr*_*a*13_ of *Bgh* (Lu et al., 2016), and *SvrPm*^*a*1/*f*1^ suppress recognition of *Avr* genes (Parlange et al., 2015; Bourras et al., 2016). A substantial number of candidate effectors from biotrophic fungal pathogens that are well-known to infect crop plants have been predicted, cloned, and characterized (Table 2). From the current studies on biotrophic plant fungal pathogen effectors, it can be concluded that, effectors play important roles in pathogenicity by subverting PTI, suppressing cell death signaling activities, promoting virulence during infection, and some of these effectors are also required for penetration and formation of infection structures such as haustoria. This clearly asserts that biotrophic plant fungal pathogen effectors employ various mechanisms to either interfere with host PTI or ETI to enhance their pathogenicity.

## Methods of Researching Effectors of Biotrophic Plant Fungal Pathogens

Genome analysis has the potential to open up new paths for the discovery of new virulence factors in biotrophic plant fungal pathogens. More information about the genome and genes related with pathogenicity can be obtained by genome sequencing. Plant-pathogen interactions studies have been transformed by genomic technologies. With the advent of next generation sequencing technology (NGS), which has reduced the cost of sequencing, a reasonable number of genomes of biotrophic fungal pathogens have recently become available. In the case of wheat rust fungi, the first pathogen genome to be sequenced was *Pgt*, which had a size of 88.6 Mb (Duplessis et al., 2011). This was followed by whole genome sequencing of *Pst*. The genome of the US *Pst* race PST-130 was the first to be sequenced and the assembled genome was 64.8 Mb in size (Table 3). A Chinese *Pst* race CYR32 provided the subsequent genome with a size of 100 Mb and a large sequencing depth (Zheng et al., 2013). Another US pathotype PST-78 provided the third genome sequence of high quality, which was 117.31 Mb in size (Cuomo et al., 2017). Three draft genome sequences have been reported for *Pt* to date including one from an isolate of extensively virulent race 77 with a size of 95.22 Mb (Kiran et al., 2016), another from an isolate of narrowly avirulent race 106 with a size of 105.07 Mb (Kiran et al., 2016) and the last from race 1 (BBBD), the most primitive race recognized in North America, with a size of 135.34 Mb (Cuomo et al., 2017; Table 3). Subsequent sequencing of five Australian *Pgt* isolates (Upadhyaya et al., 2014) and 10 *Pst* isolates from different countries was performed (Cantu et al., 2013; Zheng et al., 2013) and sequence analysis revealed large genome heterozygosity of dikaryotic rust fungi. Sequencing of the genome of the wheat powdery mildew fungus *Bgt* revealed that it is 180 Mb in size and that 7,588 protein-coding genes are reported to be encoded in this genome (Table 3). Sequencing of three other isolates, 94,202, JIW2, and 70 from Switzerland, England, and Israel respectively, was also performed and found that the *Bgt* genome exhibits a high degree of adaptability and flexibility, indicating that this biotrophic pathogen has evolved uniquely (Wicker et al., 2013). When whole genome sequencing of wheat and barley powdery mildews was analyzed, a radical decrease in gene content was observed compared to other ascomycetes, and an expansion of the putative gene complement was observed (Spanu et al., 2010; Wicker et al., 2013).

**TABLE 3:** Genomic sequencing and features of biotrophic plant fungal pathogens.

Organism	Race/isolate	Host	Disease	Genome size (Mb)	Predicted genes	Number of predicted secreted proteins	References
*Bgh*	DH14	Barley	Barley powdery mildew	87.91	7,088	248	Spanu et al., 2010
*M. larici-populina*	Isolate 98AG31	Poplar	Poplar rust	101	16,399	1,184	Duplessis et al., 2011
*Pgt*	Race SCCL	Wheat	Wheat stem rust	88.6	17,773	1,459	Duplessis et al., 2011
*Pst*	Race 130	Wheat	Wheat stripe rust	68	22,815	1,088	Cantu et al., 2011
*Pst*	CYR32	Wheat	Wheat stripe rust	130.48	25,288	2,092	Zheng et al., 2013
*Pst*	Race 31	Wheat	Wheat stripe rust	65.18	18,362	687	Kiran et al., 2017
*Pst*	PST-78	Wheat	Wheat stripe rust	117.31	19,542	Unknown	Cuomo et al., 2017
*Pst*	93-20	Wheat	Wheat stripe rust	∼89	69,513	1,517	Xia et al., 2018
*Pst*	*Pst-*104E	Wheat	Wheat stripe rust	∼83	15,928	1,069	Schwessinger et al., 2018
*M. lini*	Isolate CH5	Flax	Flax rust	189	16,271	725	Nemri et al., 2014
*Pt*	Race 77	Wheat	Wheat leaf rust	95.22	27,678	660	Kiran et al., 2016
*Pt*	Race 106	Wheat	Wheat leaf rust	105.07	26,384	620	Kiran et al., 2016
*Pt*	Race 1 (BBBD)	Wheat	Wheat leaf rust	135.34	15,539	1,358	Cuomo et al., 2017
*P. pachyrhizi*		Soybean	Soybean rust	Unknown	Unknown	851	de Carvalho et al., 2017
*P. psidii*	PBI	Myrtaceae	Myrtle rust	103–145	Unknown	Unknown	Tan et al., 2014
*P. coronata*	12SD80	Oat and barley	Oat and barley crown rust	99.16	17,248	1,532	Miller et al., 2018
*P. sorghi*	*Ps* RO10H11247	Maize	Maize common rust	99.53	21,087	1,599	Rochi et al., 2018
*P. hordei*	Ph560	Barley	Barley leaf rust	207	25,543	1,450	Chen et al., 2019
*P. arachidis*	MRf11	Groundnut	Groundnut/Peanut rust	87.68	Unknown	Unknown	PRJNA280565
*P. horiana*	SC2014G01	Chrysanthemum	Chrysanthemum white rust	66.4	Unknown	Unknown	PRJNA306202
*Hemileia vastatrix*	XXXIII (Hv33)	Coffee	Coffee leaf rust	547	143,364	615	Porto et al., 2019

During plant-pathogen interactions, the expression profiles of secreted proteins are characteristically complicated. Fungal structural differentiation, competing microbes, and cell-to-cell communication secrete various non-effectors that are essential for the colonization and fortification of biotrophic plant fungal pathogens. Consequently, the search for suitable effectors for subsequent experimental validation is economical for ensuing experimental corroboration. Discovery of biotrophic plant fungal effectors is classically performed using an integration of experimental techniques like genome-wide association study (Sánchez-Vallet et al., 2018; Richards et al., 2019), comparative genomics and phenotype association (Williams et al., 2016; Plissonneau et al., 2017; Wu et al., 2017, 2020; Beckerson et al., 2019; Chen et al., 2019; Mousavi-Derazmahalleh et al., 2019), proteomics (Gawehns et al., 2015; Mesarich et al., 2018), and transcriptomics (Gervais et al., 2017; Jones et al., 2019; Elmore et al., 2020; Human et al., 2020). Recently developed machine learning algorithms for detecting proteins with effector-like features have opened up new avenues for refining effector prediction pipelines. FunEffector-Pred (Wang et al., 2020) and EffectorP (Sperschneider et al., 2016, 2018b) utilize amino acid molecular weight, charge, frequencies, and other protein features to directly predict effector-like proteins. Tools like EffectorP and FunEffector-Pred, when combined with secretion prediction, may provide a more robust alternative to basic hard filters. Signal peptides of effectors can be predicted using some online searches. These tools involve integration of various taught algorithms and are usually very sensitive and accurate (Sonah et al., 2016). Since both transmembrane proteins and effector proteins contain hydrophobic segments, it is of great importance to distinguish the two and effectors have been found to contain a shorter hydrophobic portion compared to transmembrane proteins. Various online tools and WEB servers have been used for transmembrane domain prediction. These include TMHMM server v.2.0, and others (Sonah et al., 2016). YLoc, WoLF PSORT, Phobius, and SignalP are very helpful in predicting signal peptides as well as extracellular localization of candidate effectors. Prediction of the localization of candidate effectors from the plant cytoplasm, nucleus, mitochondria, and chloroplasts has been greatly improved by the development of the tools ApoplastP (Sperschneider et al., 2017; Sperschneider et al., 2018a) and LOCALIZER (Sperschneider et al., 2018b), and these tools are also beneficial for evaluating candidates but do not always predict effector candidature (Jones et al., 2021). However, computer-based prediction tools do not provide concrete results because they focus on a few features such as sequence similarities or small size. Therefore, it is necessary to include additional materials such as *in-planta* expression data and any other relevant information to improve the predictability of effectors of a pathogen of concern. The increased integration of next-generation sequencing and high-throughput genotype technologies has highly accelerated the identification of candidate secreted effector proteins (CSEPs) encoding Avr proteins in powdery mildews (Bourras et al., 2018). Bulk segregant analysis (BSA) (Bourras et al., 2015), map-based cloning, genome-wide association studies (GWAS) (Praz et al., 2017), and RNAseq based GWAS (Lu et al., 2016) were used in the identification of powdery mildew *Avrs* (Bourras et al., 2018). These methods were used in the cloning and characterization of four avirulence effectors: *AvrPm3*^*a*2/*f*2^, and *AvrPm2* from *Bgt* which are recognized by *Pm3a/f*, and *Pm2* wheat *R* genes, respectively (Bourras et al., 2015; Parlange et al., 2015; Lu et al., 2016; Praz et al., 2017), and *Bgh Avr*_*a*1_ *and Avr*_*a*13_ which are recognized by *Mla1* and *Mla13* barley *R* genes respectively (Lu et al., 2016), and also *SvrPm*^3*a*1/*f*1^ which suppresses Avr recognition (Bourras et al., 2015; Parlange et al., 2015). Due to the complexity of rust structure and large genome, RNA-seq is considered an effective method for identifying CSEPs. 16,399 and 17,773 secreted proteins were found from the sequencing of *Mlp* 98AG31 and *Pgt* SCCL transcriptome, respectively (Duplessis et al., 2011). Sequencing of differential virulence stripe rust races PST-87/7 and PST-08/21 from United Kingdom, identified only five candidate effector proteins with polymorphism among 2,999 secreted proteins (Cantu et al., 2013). Six different *Pt* races that interacted with wheat, 6 days post-inoculation were sequenced using Illumina platform and 532 secreted protein candidates were predicted, of which 456 were present in the assays for all races, and 12 *Avr* candidate genes were predicted (Bruce et al., 2014). The efficiency in predicting CSEPs is improved by integrated software that includes EffectorP v2.0, SignalP v4.1, TMHMM v2.0, and TargetP v1.1 for the analysis of RNAseq data. Recently, Zhang and colleagues used these softwares to screen CSEPs in wheat *Pt* KHTT, THSN, and JHKT isolates and obtained a total of 635 candidate effector proteins with small size (50-422 amino acids) and different sequences. *Pt* CSEPs were found to possess various family domains including thaumatin family, glycosyl hydrolase family, Barwin family, and others, and different motifs were also detected, such as RxLR, YxSL[R/K], [L/I]xAR, [Y/F/W]xC, G[I/F/Y] [A/L/S/T]R, and three *de novo* motifs (Zhang et al., 2020). For the identification of genes involved in *Pt* pathogenicity and virulence, Wei et al. (2020) performed RNA-seq at 144hpi to analyze the differentially expressed genes between THTT and THTS *Pt* pathotypes, to gain more insight into the molecular basis of *Pt-*wheat interaction. mRNA was sequenced and a total of 2,784 DGEs were detected, of which 45 genes were specifically expressed in THTS and 44 differentially expressed effector candidates were observed in both isolates. Analysis of the obtained results indicated that, although the two pathotypes of THTT and THTS contribute similar virulence to wheat, many genes are involved in the interaction with susceptible wheat cultivar Thatcher. This also demonstrates that the pathogenicity of rust is very complicated (Wei et al., 2020). In another recent study, genome-wide association was used in profiling of effector candidate associated with the infection process of the *Pt* pathotype PHTTP (P). Zhao et al. (2020) established that, 363 secreted proteins were encoded by upregulated genes, with only 79 of them also predicted as possible effectors by EffectorP. Using Regex and hmmsearch, 719 RXLR-like, 138Y/F/WxC, 19PNPi-like, 19CRN-like, and 9 CFEM effector candidate were identified from the deduced database, including transcriptome data from this study and data based on the Pt 1-1 BBBD Race 1 genome. Of the 19 PNPi-like candidate effectors, four of them possessing DPBB_ 1 conserved domain demonstrated physical interactions with wheat NPR1 protein in yeast two-hybrid assay. Only seven CFEM and nine Y/F/WxC candidate effectors were transiently expressed in *Nicotiana benthamiana*. None of the predicted effector candidates showed suppression or induction of cell death triggered by the protein BAX, but acceleration of cell death and enhanced ROS accumulation was only shown by the expression of PTTG_08198, a CFEM effector candidate (Zhao et al., 2020). AvrSr35, AvrSr50, and AvrSr27 were cloned, and mutation, RNA-seq and genome sequencing were performed on *Pgt* isolates (Chen et al., 2017; Salcedo et al., 2017; Upadhyaya et al., 2021). AvrSr35, AvrSr50, and AvrSr27 discovery did not only provide new tools for identifying *Pgt* avirulence gene and characterizing the molecular determinants of immunity in wheat, but also revealed the complexity of *Pgt* pathogenicity mechanism.

A comparative genomics approach was integrated with association analysis to identify candidate effector genes corresponding to *Lr20* in phenotypically paired Australian *Pt* isolates. This study employed whole-genome sequencing to analyze twenty *Pt* isolates consisting of 10 phenotype-matched pairs with different *Lr20* pathogenicity. This was the first study to integrate phenotype-genotype associations with effector prediction in *Pt* genomes and the approach used could circumvent the technical difficulties of working with obligate *Pt* and accelerate the identification of avirulence genes (Wu et al., 2017). Wu et al. (2020) carried long-read-based *de novo* genomy assembly and comparative genomics of wheat leaf rust pathotype Pt104 and identified candidates for avirulence genes. They established that *AvrLr2a* canditae GN104ID162_007386, *AvrLrka* candidate GN1041D162_024924, and the *AvrLr26* candidate GN104ID162_020918 had corresponding orthologs of PTTG_07365, PTTG_28070, and PTTG_1194 in the *Pt* race 1 genome, respectively (Wu et al., 2020). In support of their findings, a proteomics study of *Pt* race 1 haustoria predicted that these *Pt* race 1 orthologs were also candidate effectors (Rampitsch et al., 2015).

The use of natural pathosystems to confirm biotrophic fungal effectors has been challenging, leading to the use of alternative hosts for their expression. This is now the most economical, rapid and applicable approach to validate biotrophic fungal effectors. In addition, *N. benthamiana* has been found to be an appropriate expression system for the obligate biotrophic fungal pathogens, particularly for validating rust effector proteins. This has also been successfully used in the identification of rust effectors acting at many sites in the plant cell compartments. In a recent study, the transient expression system of mesophyll protoplasts was found to be an important and useful tool for studying signal transduction in pathogen-model plant interactions (Su et al., 2021). Chitin and flg22 can be used as triggers for the PTI response in wheat leaves and TaPr-1-14 can also be used as a marker gene for detecting the PTI response. Screening and identification of *Pst* effector proteins can be performed rapidly and efficiently using a transient wheat protoplast expression system (Su et al., 2021). Out of 39 haustorial effector genes that were successfully screened and cloned using the protoplast transient expression system, three haustorial effector proteins PSEC2, PSEC17, and PSEC45 were identified and had the ability to inhibit wheat response to PTI. The three effector proteins were highly expressed during wheat infection and parasitism and were found to be localized in wheat protoplast somatic cytoplasm and nucleus (Su et al., 2021). The number of effectors identified recently has been greatly accelerated by access to fungal transcriptomic and genomic sequences. The triumph in identifying effectors could envisage their virulence-promoting or host-manipulating function, but, their exact function can only be guessed after identifying their interacting partners. Various approaches including co-immunoprecipitation (Co-IP) assays, yeast-two-hybrid (Y2H) approaches and pull-down assays of the target can be used to validate effector proteins and their interacting targets (Prasad et al., 2019; Jaswal et al., 2020). Advances in genomics studies paved a way for the identification of a repertoire of candidate effectors while some computational approaches coupled with high-throughput tools continue to help in the functional characterization of the effectors. A comprehensive understanding of pathosystems effector biology and the breeding of resistant crops has been facilitated by the discovery of a repertoire of effectors (Prasad et al., 2019).

## What Needs to Be Improved in Studying Biotrophic Plant Fungal Pathogens

The study of biotrophic pathogens has made considerable progress following the advent of the genomics era. However, most disease-causing organisms that grow on plants or have a reciprocal relationship with the host cannot be cultured. The lack of an effective genetic transformation system has hindered the progress in understanding the pathogenicity mechanisms of the biotrophic fungi. Little is known about how the biotrophic fungal pathogens absorb their nutrients from living host cells, and the genetic factors that determine the host exclusivity. How are the effector proteins delivered into the host cytoplasm and what is the functional compartment of the effectors? What is the target in the host? What affects the signal system pathway? A detailed answer to these questions is required to understand the pathogenicity mechanisms of biotrophic plant fungal pathogens. The mining and identification of candidate effectors and related genes are important first step toward functional assays to decipher their contribution to pathogenicity. The main research methods used in the study of biotrophic plant fungal pathogens include genome sequencing, transcriptome analysis, software analysis, and heterologous expression. Although they are more efficient than ever, they need to be improved, especially the need to develop a highly effective genetic transformation system. The establishment of a genetic transformation system for biotrophic fungi will greatly promote the study of pathogenic mechanisms of obligate parasites and the application of the corresponding genes. In order to broaden the toolbox to study biotrophic plant fungal pathogens, new methodologies and techniques such as for the visualization of plant-pathogens interactions must be developed. Rust–plant interactions still require effective techniques for unraveling their specific molecular mechanisms. Standardized routines in genomics needs to be developed as well as constructing functional pipelines to improve comparative studies. New techniques must be developed for the study of the dynamics of biotrophic interactions, such as metabolic pathways and fluxes, *in situ.*

## Concluding Remarks

Biotrophic plant fungal pathogens differ from the necrotrophs and hemi-biotrophs in their infection strategies and pathogenicity mechanisms, and are currently the focus of scientific research in molecular plant pathology because their sophisticated infection strategies make them a constant threat to many crops around the world. Normally, they require specialized infection structures to avoid host recognition and to facilitate their attachment and penetration. They also require a limited amount of enzymes to promote pathogen penetration and infection. The haustorium is the most important structure. Haustoria are considered not only as means of nutrition acquisition for the invasive growth of biotrophic plant fungal pathogens but also as an arsenal of effectors to bypass the host surveillance system and undermine the host’s multilayered defenses. The molecular bases of obligate biotrophy have been revealed by comparative genomic analyzes and transcriptome analyzes and these may pave the way for establishing biotrophic *in vitro* culture protocols for biotrophic plant fungal pathogens by imitating the absorption state of nutrients. Biotrophic plant fungal pathogens effectors are fundamental for establishing compatible interactions with the host plant. Moreover, they are undoubtedly virulence factors that are secreted to suppress or regulate host defenses by targeting host cognate proteins associated with resistance and modulating host cellular responses, thereby promoting virulence and pathogenicity. Due to the parasitic nature of biotrophic plant fungal pathogens, the lack of an effective transformation system affects the analysis of the functions of pathogenic fungi effector proteins and pathogenic genes. However, the current heterologous expression system and host-induced gene silencing technology still have some targeting and genetic background effects, which may affect the accurate analysis of the biological function of genes. Therefore, it is necessary to develop an efficient transformation system in the future to provide a good approach for gene function analysis of the biotrophic pathogenic fungi. Understanding effectors provide a fascinating illustration of the remarkably sophisticated molecular dialog that exists between biotrophic plant fungal pathogens and their hosts. By integrating genomics, transcriptomics and effectoromics, insights into the adaptation of biotrophic plant fungal pathogens to their host populations can be gained. Functional characterization of effectors during host-pathogen interactions will provide the basis for future studies on the role of effectors and haustoria, for understanding pathogenesis mechanisms, and for developing effective management strategies to control diseases caused by pathogenic rust fungi and powdery mildew.

## Author Contributions

JM and WY conceptualized the manuscript, went through all the research manuscripts, and finalized the manuscript. JM researched on the topic and wrote the review. All authors revised the manuscript and approved it for publication.

## Conflict of Interest

The authors declare that the research was conducted in the absence of any commercial or financial relationships that could be construed as a potential conflict of interest.

## Publisher’s Note

All claims expressed in this article are solely those of the authors and do not necessarily represent those of their affiliated organizations, or those of the publisher, the editors and the reviewers. Any product that may be evaluated in this article, or claim that may be made by its manufacturer, is not guaranteed or endorsed by the publisher.

## Abbreviations

Avr: avirulence
AC: adenylate cyclase
CSEPs: candidate secreted effector proteins
DAMPs: damaged-associated molecular patterns
ETI: effector-triggered immunity
PAMPs: pathogen-associated molecular patterns
PCD: programmed cell death
PRRs: pathogen recognition receptors
PTI: pathogen-associated molecular pattern triggered immunity
ROS: reactive oxygen species.
